# Black Elderberry Press Cake as a Source of Bioactive Ingredients Using Green-Based Extraction Approaches

**DOI:** 10.3390/biology11101465

**Published:** 2022-10-06

**Authors:** Zorana Mutavski, Nataša Nastić, Jelena Živković, Katarina Šavikin, Robert Veberič, Aljaž Medič, Kristian Pastor, Stela Jokić, Senka Vidović

**Affiliations:** 1 Faculty of Technology Novi Sad, University of Novi Sad, 21000 Novi Sad, Serbia; 2 Institute for Medicinal Plants Research “Dr Josif Pančić”, 11000 Belgrade, Serbia; 3 Biotechnical Faculty, University of Ljubljana, SI-1000 Ljubljana, Slovenia; 4 Faculty of Food Technology Osijek, University of Josip Juraj Strossmayer in Osijek, 31000 Osijek, Croatia

**Keywords:** elderberry press cake, *Sambucus nigra*, anthocyanins, ultrasound-assisted extraction, microwave-assisted extraction, antidiabetic activity

## Abstract

**Simple Summary:**

Current practice in the management of fruit waste and by-products does not fully exploit waste potential, since fruit waste often contains valuable compounds that can be extracted and used in different industries. Black elderberry press cake is a by-product of the production of black elderberry juice that contains residual polyphenolic compounds with different bioactive potentials. The main focus of this research was to propose new streams to use black elderberry press cake, specifically to demonstrate the possibility of implementing green and safe technology in the food and pharmaceutical industries in order to enable more economical waste management, as well as to develop new technological lines that will increase economic growth. Our findings provide comprehensive approaches for treating anthocyanin-rich black elderberry press cake, which is highly correlated with antidiabetic potential, thereby enhancing its application for the development of novel food and herbal formulations.

**Abstract:**

To study the efficiency of two green-based extraction techniques for the isolation of bioactive constituents from black elderberry press cake, changes in phenolic compounds and main anthocyanin contents were analyzed. Polyphenolic content was correlated with antioxidant and antidiabetic potential by radical-scavenging activity and monitoring of *α*-amylase inhibition. Black elderberry press-cake extracts were obtained by ultrasound-assisted (UAE) and microwave-assisted (MAE) extractions under different extraction conditions. High-performance liquid chromatography (HPLC) analysis revealed that cyanidin-3-sambubioside and cyanidin-3-glucoside were the principal anthocyanins in all the obtained extracts, with their content being highest in MAE obtained at 80 °C over 5 min. The same extract induced two-fold higher antioxidant activity (IC_50_ 6.89 μg/mL) and α-amylase inhibitory potential (IC_50_ 2.18 mg/mL) in comparison to UAE extracts. The main compositional differences between extracts obtained by the same extraction technique were assigned to the extraction temperature. A principal component analysis confirmed that the antidiabetic feature is to be attributed to the rich content of anthocyanins in black elderberry press cake. Our results indicate the great potential of underutilized black elderberry press cake for the development of novel food and herbal formulations due to notable anthocyanin contents highly correlated with antidiabetic activity.

## 1. Introduction

In contemporary society, the human population is turning to use natural products due to the increased harmful effects caused by various synthetic additives [1]. The use of fruit- and vegetable-based products has become part of a model of healthy living and health maintenance. On the other side, producers must meet their target groups’ needs. A large amount of waste and by-products (about 33% of the initial fruit mass) is generated in the production of fruit and vegetable juices [2]. The question is whether it is possible to use these wastes and by-products after industrial production and how to use them in order to obtain some valuable nutrients or bioactive compounds. The goal of full utilization of raw material is to monitor the zero-waste concept and to establish production for maximum utilization [3].

Black elderberry (*Sambucus nigra* L.) press cake (a by-product left after juice processing) has been profiled as an efficient and alternative bioresource for various nutrients and bioactive phytochemicals, such as phenolics, anthocyanins, etc. Using different valorization approaches which can optimize the processes, the development of procedures for green waste sustainability and consequent economic benefits could be achieved. According to statistical data published by the Food and Agriculture Organization (FAO) [4], 43,804 tons of fresh fruit were produced in Europe in 2020, including elderberries. The high demand for elderberry-based products and dietary supplements has led to increased elderberry production over the last several years, mostly due to the COVID-19 pandemic [5,6]. The annual growth rate of the elderberry market is forecast to be 6.52% by 2025, which will generate a large amount of waste after elderberry processing and open possibilities for its utilization [7]. Elderberries are mostly composed of hemicellulose and cellulose, but they also represent an important source of polyphenolic compounds. Recent studies have reported that most of the polyphenols and anthocyanins in black elderberry remain in the press cake after juice pressing. Among the anthocyanins, the most abundant are cyanidin-3-sambubioside and cyanidin-3-glucoside [8]. Plant-based products rich in anthocyanins are believed to have pharmacological relevance and therapeutic applicability due to their biological activities, especially antioxidant effects. Anthocyanins can reduce neurological diseases and heart-disease risks, and exert an anti-inflammatory role related to obesity and diabetes [9,10]. Anthocyanins have been described in the literature as inhibitors of *α*-amylase. They also regulate blood glucose, normalizing insulin secretion and resistance [11]. However, despite its high nutritional and pharmacological values and the economic interest in bioactive substances, black elderberry press cake is an underutilized resource.

However, in recent years, thanks to new scientific cognitions related to mechanisms enabling the separation of compounds from complex matrices, significant progress has been made in developing technology for the isolation of pharmacologically active compounds of natural origin. Special attention has been paid to the development of green chemical processes with reduced extraction times using environmentally friendly solvents. Furthermore, the use of these techniques reduces the generation of new waste through striving after the full utilization of resources [12,13]. Ultrasound-assisted extraction (UAE) and microwave-assisted extraction (MAE) are emerging techniques increasingly used in many industries. The ultimate effect of these techniques is to destroy the cell walls of plants and release phytochemical compounds into solvents [14]. According to the literature, MAE was previously performed on elderberry press cake to isolate pectin from defrosted raw material [15]. In a study by Kitrytė et al. (2020) [16], UAE was used to isolate non-polar compounds and sambunigrin from black elderberry press cake, using hexane as a solvent. These data enabled the expansion of UAE and MAE applications for elderberry press-cake valorization. The mentioned techniques were also applied in the isolation of polyphenolic compounds, primarily anthocyanins, from dried elderberry fruit [17,18,19].

For the recovery of these valuable compounds from elderberry press cake, also considering safety, the low costs, selectivity and the solvating properties of solvents, this work investigated the possibilities of applying green extraction techniques. To our knowledge, this is the first study that has been carried out to recover phenolic compounds from elderberry press cake and produce anthocyanin-enriched extracts using novel green extraction technologies (UAE and MAE). Characterizations of polyphenolic compounds and the influence of different extraction parameters on the recovery of polyphenolics from elderberry press cake were also determined. Correlations between the contents of individual anthocyanins and flavonoids and their antidiabetic and antioxidant activities were evaluated. Overall, the main goal was to prove that the applied novel extraction techniques are more effective for the isolation of polyphenolic compounds from black elderberry cake and that the obtained compounds are positively correlated with the antioxidant and antidiabetic activities of the obtained extracts.

## 2. Materials and Methods

### 2.1. Plant Material and Chemicals

Fresh black elderberry press cake (*Sambucus nigra*) was provided by a factory focusing on harvesting and processing forest fruits (NISHA d.o.o., Belgrade, Serbia). The raw material was dried using a vacuum dryer and ground in a blender. The average particle size (1.05 ± 0.16 mm) and moisture content (6.69 ± 0.13%) were determined. The material was then stored in a desiccator in the dark at an ambient temperature.

Folin–Ciocalteu reagent, gallic acid, (+)-catechin, acarbose, 2.2-diphenyl-1-picrylhydrazyl (DPPH), amylase (from the porcine pancreas) and formic acid were purchased from Sigma-Aldrich (Steinheim, Germany). Ethanol (EtOH) and methanol (MeOH) were purchased from Centrohem Šabac (Šabac, Serbia). Acetonitrile (Merck, Germany) was of HPLC grade and ultrapure water was prepared using a Milli-Q purification system (Millipore, France). All of the standard anthocyanin compounds (grade purity >96%) and flavonoids (grade purity >99%) were purchased from Extrasynthese (Cedex, France). All other chemicals were of analytical grade and used without further purification.

### 2.2. Conventional Solid–Liquid Extraction (SLE)

In all of the SLE experiments, 5 g of plant material was extracted with 50 mL (1:10) of different concentrations of EtOH (30, 50 and 70%; *m*/*m*). Extractions were performed at 25 °C for 24 h. After extraction, the extracts were filtered through filter paper under vacuum conditions, then transferred to vials and stored at 4 °C until analysis.

### 2.3. Ultrasound-Assisted Extraction (UAE)

UAEs were conducted using an ultrasound probe (Hielscher Ultrasonic GmbH, Germany), with amplitudes of 20, 60 and 100% and varying temperatures of 40, 60 and 80 °C. An amount of 10 g of plant material was mixed with 100 mL of 30% EtOH as a solvent (solid–liquid ratio 1:10). Mixing was performed with a magnetic stirrer (IKA RH basic 2, IKA, Staufen, Germany) at 300 rpm. The extraction time was defined as the time required to reach the set temperatures, and, for that reason, it was different for all extractions (1–40 min). After extraction, the extracts were filtered under a vacuum, collected in vials and stored at 4 °C until analysis.

### 2.4. Microwave-Assisted Extraction (MAE)

MAEs were performed using a Milestone flexiWAVE Microwave apparatus and a closed-vessel system (Milestone Srl, Sorisole (BG), Italy), with the maximum microwave power set at 1800 W. Briefly, 10 g of plant material was mixed with 100 mL of 30% EtOH as a solvent. This technique was performed at five different temperatures (40, 60, 80, 100 and 120 °C), for 5 and 10 min. In order to maintain a constant temperature, the power was not applied constantly during the experiment. The pre-heating time was set for 1 min and the cooling time for 10 min. The obtained extracts were filtered and collected in vials, then stored at 4 °C until analysis.

### 2.5. Extraction Yield (EY) and Total Phenolic (TPC) and Flavonoid Contents (TFC)

The EY was expressed as the mass of collected extract (g) per gram of dry plant material, i.e., percentage (%). 

TPC was determined using the Folin–Ciocalteu procedure [20]. Fruit extracts were dissolved in methanol. In this method, the fruit extract (0.1 mL), distilled water (7.9 mL), Folin–Ciocalteu reagent (0.5 mL) and saturated Na_2_CO_3_ solution (1.5 mL) were added to a test tube. A control sample was prepared at the same time using distilled water (8 mL), Folin–Ciocalteu reagent (0.5 mL) and saturated Na_2_CO_3_ solution (1.5 mL). Ingredients in test tubes were well mixed using the Vortex and left in a dark place for 2 h. The absorbance of the samples was measured at 750 nm (6300 Spectofotometer, Jenway, UK). TPC was expressed as milligrams of gallic acid (GAE) per gram of dried extract (DE). 

TFC was determined using the aluminum chloride (AlCl_3_) spectrophotometric assay [21]. An aliquot of extract (1 mL) was added to a volumetric flask containing a solution of NaNO_2_ (0.3 mL, 0.5 g/L). After 5 min, a 1 g/L solution of AlCl_3_ (0.3 mL) was added and, 6 min later, NaOH (2 mL, 1 mol/L) was added to the mixture. The total volume was made up to 10 mL with distilled water, the solution was mixed, and the absorbance was measured at 510 nm. TFC was expressed as milligrams of (+)-catechin (CE) per gram of dried extract (DE).

### 2.6. HPLC-MS/MS Analysis

Phenolic compounds were analyzed at 290 nm (phenolic acids), 350 nm (flavanols and flavonols) and 520 nm (anthocyanins). The column used was a Gemini C18 (150 × 4.60 mm, 3 μm; Phenomenex, Torrance, CA, USA), operated at 25°C. The mobile phase consisted of solvent A (10%, *v*/*v* solution of formic acid in water) and solvent B (acetonitrile), and the samples were eluted using the same gradient as in the case of HPLC-DAD analysis. The injection volume was 10 μL, and the flow rate was maintained at 1 mL/min. All phenolic compounds in the elderberry products were identified with a mass spectrometer (LCQ Deca XP MAX, Thermo Fisher Scientific, Waltham, MA, USA) with electrospray ionization (ESI) operated in positive-ion mode for anthocyanins and in negative-ion mode for the remaining compounds. The analyses were carried out using full-scan data-dependent MS^n^ scanning from *m*/*z* 110 to 1600.

### 2.7. HPLC-DAD Analysis

Analyses of individual polyphenolic compounds were carried out on an Agilent 1200 RR system (Agilent, Waldbronn, Germany) with a diode array detector. A reverse-phase Lichrospher RP-18 (Agilent) column (250 mm × 4 mm, 5 μm) was used, and the column temperature was maintained at 25°C. The mobile phase consisted of solvent A (10%, *v*/*v* solution of formic acid in water) and solvent B (acetonitrile), using gradient elution as follows: 1% B, 0–0.5 min; 1–7% B, 0.5–1 min; 7% B, 1–4 min; 7–10% B, 4–7.5 min; 10–14% B, 7.5–11.5 min; 14–25% B, 11.5–15.5 min; 25–40% B, 15.5–18.5 min; 40–75% B, 18.5–22 min; 75% B, 22–25 min. The injection volume was 10 μL, the flow rate was 1 mL/min and the detection wavelengths were set at 290, 350 and 520 nm. The contents of the compounds were calculated using calibration curves. The results are presented as milligrams per gram of dried extract (mg/g DE). Of the detected polyphenolic compounds, eight of them were quantified by measuring peak areas against a standard curve constructed with commercial standards, including: cyanidin-3-galactoside, cyanidin-3-sambubioside (calculated based on a standard curve for cyanidin-3-glucoside), cyanidin-3-glucoside, kaempferol derivative 1, kaempferol derivative 2 (both calculated based on a standard curve for kaempferol) and chlorogenic acid.

### 2.8. DPPH Radical Scavenging Activity

The antioxidant activities of the liquid extracts were analyzed using the DPPH assay, as described by Espín et al. (2000) [22]. Different volumes (50–125 μL) of diluted sample/liquid extract were mixed with 95% MeOH and 90 μmol/L DPPH solution to obtain different final concentrations of the test sample. For the control, 30% EtOH was used instead of extract solution. The absorbance of the test sample and control was measured at 515 nm after 60 min of incubation at room temperature. The antioxidant activity obtained by the DPPH method was first expressed as radical-scavenging capacity (RSC) and further as the IC_50_ value, where IC_50_ represents the concentration of the test sample that inhibits 50% of DPPH radicals present and which is required to obtain 50% RSC.

### 2.9. Determination of α-Amylase Inhibitory Activity

Determination of *α*-amylase inhibition activity was carried out according to the method of Šavikin et al. (2018) [23], with minor modifications. Briefly, 200 μL of diluted extracts were mixed with 200 μL of 0.10 mg/mL *α*-amylase enzyme solution. Solutions of extracts and enzymes were prepared in phosphate-buffered solution (0.1 M, pH 6.9). After 15 min of incubation at 37 °C, 200 μL of 1% (*m*/*v*) starch dissolved in phosphate buffer was added, and incubation was continued for another 10 min at 37 °C. After that, 200 μL of dinitrosalicylic acid reagent was added and the samples were placed in a boiling water bath for 15 min. Absorbance was measured at 540 nm. Acarbose was used as a positive control. The results were expressed as sample concentrations that led to a 50% reduction in *α*-amylase activity (IC_50_).

### 2.10. Data Processing

All analyses were carried out in triplicate, and the results were expressed as means ± standard deviations (SDs). Mean values were considered significantly different at the *p* < 0.05 confidence level. The performance of one-way ANOVA statistical analysis was followed by Tukey testing.

Principal component analysis (PCA) is a widely used tool in food and natural-product research. The method has been employed to develop techniques for flour [24] and honey authentication [25]. The effect of deep-frying potato and tofu on thermo-oxidative changes in cold-pressed rapeseed oil, high-oleic rapeseed oil and palm olein was investigated by Wroniak et al. [26], and a volatile profile for Kumpiak podlaski dry-cured ham during traditional ripening was developed by Karpinski et al. [27].

In order to investigate the relationships between the identified polyphenolic compounds and antidiabetic and antioxidant properties on one side and the different extraction conditions on the other, within a multivariate data space, principal component analysis (PCA) and Pearson’s correlation analysis were applied [24]. PCA is one of the most common multivariate data analysis tools for unsupervised dimensionality reduction and pattern recognition. Its main aim is to find the PCA space, which represents the direction of the maximum variance of the given data [28]. The absolute values of compound concentrations in milligrams per gram of dried extract (DE), expressed as means of three repetitions, were used as inputs for creating PCA biplots. Data analyses were performed using Paleontological Statistics Software PAST v4.10 (Natural History Museum, University of Oslo, Norway).

## 3. Results and Discussion

### 3.1. Determination of Extraction Efficiency

The conventional SLE was applied as a reference technique to select the optimal extraction solvent. As regards SLE, EYs ranged from 13.92 to 26.58% (Table 1). The highest EY was obtained by extraction with 30% EtOH (26.58%), which followed the highest measured TPC for the extract of 150.50 mg GAE/g DE. By measuring the TFC, a different trend was observed. Increasing the concentration of EtOH, the TFC slightly increased. SLE with 70% EtOH proved to be the most efficient for flavonoid extraction (49.51 mg CE/g DE). Based on the obtained data, 30% EtOH was selected as the optimal solvent to be used for UAE and MAE.

UAEs were performed at three different sonication amplitudes: 20, 60 and 100%. The extraction time was defined as the time required to reach the set temperatures (40–80 °C), and, for that reason, it was different for all extractions (Table 1). Table 1 shows that, at an amplitude of 20%, extractions were performed only up to 60 °C. The extraction time required to achieve a higher temperature would be too long, making the system economically unviable when compared with other techniques and conditions. In all UAEs, EYs ranged between 19.68 and 31.28%. The highest EY was achieved when the extraction was performed at 80 °C with an amplitude of 100% (UAE-13), while the lowest applied temperature and amplitude exhibited the lowest EY. The thermal effect increases with amplitude, since at high amplitudes the number of compression and rarefaction cycles of ultrasonic waves increases, leading to an intensification of cavitation-induced phenomena [29]. An increase in extraction temperature is expected to promote extraction efficiency, due to tissue softening and a concomitant increase in diffusion rate [30]. The same effect of temperature on EY has been reported in several studies [31,32]. If we observe the values from Table 1 for TPC, it is to be concluded that the highest TPC was achieved at an amplitude of 60% with a temperature of 60 °C (143.23 mg GAE/g DE) (UAE-6). As for flavonoids, the most optimal conditions for their isolation were the conditions under which UAE-9 was obtained, namely, 40 °C and an amplitude of 100%. It was observed that an increase in temperature, as well as an increase in the sonication amplitude, favorably affected EY and TPC up to a certain value, after which the values decreased due to the increase in solvent viscosity and the decomposition of certain compounds [33]. This can justify the highest TPC in the UAE-6. Duymuş et al. (2014) [34] obtained values for TPC in the range of 49.17–89.74 mg GAE/g DE in dried elderberry fruits extracted by SLE using different extraction solvents (water, 70% ethanol and MeOH). In addition to the obtained lower TPC, the extraction time (48 h) was considerably longer in comparison to the maximum of 40 min for UAE-1. The authors also carried out UAE with methanol as a solvent, achieving a two-fold lower TPC (63.99 mg GAE/g DE) than the lowest TPC value observed in this research.

As with UAE, the extraction efficiency of MAE was also evaluated. Using a temperature of 120 °C, during 10 min of extraction, the highest EY was obtained (25.77%), while the lowest EY was obtained by applying 60 °C for 5 min. During MAE for 10 min, the increase in extraction temperature resulted in an increase in EY. A similar trend was observed for MAE during 5 min, except for MAE-1 (40 °C). Such a thermal effect was expected, since an increase in this parameter is known to increase the energy amount delivered to the treated sample [35,36]. The highest TPC and TFC values were achieved with the MAE-2 extract (166.02 mg GAE/g DE and 54.30 mg CE/g DE, respectively). Radványiet al. (2013) [37] investigated the efficiency of the extraction of polyphenolic compounds from elderberry press cake, using EtOH/water as a solvent at different concentrations (0–90%). The highest EY (15.2%) was obtained with the aqueous extract, this being two-fold lower in comparison with the maximum values obtained for each extraction technique considered in this research. Slightly lower EYs were reported in the study conducted by Seabra et al. (2010) [38], where fractionated, high-pressure extractions from elderberry press cake were performed using supercritical CO_2_ extraction, followed by enhanced solvent extraction (ESE) with diverse CO_2_/ethanol/H_2_O solvent mixtures. All these results show the advantages of the application of UAE and MAE as green-based approaches for the valorization of black elderberry press cake.

### 3.2. HPLC Analysis of Phenolic Compound in Obtained Extracts

The phenolic compounds in black elderberry press-cake extracts were identified and quantified by HPLC-MS/MS and HPLC-DAD analysis. The polyphenolic profile of black elderberry press-cake extracts is presented in Table 2 and Figure S1 (Supplementary Materials). A total of 16 polyphenolic compounds were detected in the obtained extracts, seven of which were identified for the first time.

Four compounds, cyanidin-3-galactoside, cyanidin-3-sambubioside, cyanidin-3-glucoside and cyanidin-3-sambubioside derivative, were elucidated as anthocyanins. Other compounds belonging to the flavonoids were also extracted, namely, flavanols and their derivatives, rutin, isoquercitrin, kaempferol derivative 1 and 2, kaempferol-3-rutinoside, and one flavonol derivative, quercetin trisaccharide. Concerning the hydroxycinnamic derivatives, chlorogenic acid, ferulic acid hexoside derivative 1 and 2, *p*-coumaric acidhexoside derivative and dicaffeoylquinic acid derivative were identified, and one hydroxybenzoic acid, protocatechuic acid. To the best of our knowledge, only a few reports have been published that have dealt with polyphenolic compounds in black elderberry press cake [37,38,39,40]. However, this is the first report with a more comprehensive analysis of the polyphenolic composition of black elderberry press cake.

For all of the quantified compounds, MAE exhibited the highest recoveries compared to the other methods (Table 3). Two-fold lower recoveries were achieved by UAE, while SLE proved to have the poorest recovery for all these compounds. The recoveries of cyanidin-3-galactoside, cyanidin-3-sambubioside, cyanidin-3-glucoside, rutin and kaempferol derivative 1 were found to be highest in the extracts obtained by MAE at 80 °C over 5 min (MAE-3), while isoquercitrin and chlorogenic acid were extracted better by the same technique but at a lower temperature (MAE-2). MAE-9 at 100 °C over 10 min was the richest in kaempferol derivative 2. The application of microwaves in combination with a higher temperature and a prolonged time (120 °C, 10 min) resulted in a lower extraction efficiency, as the concentrations of all compounds in the produced extracts were reduced by approximately two to ten times, indicating the possible degradation of thermolabile phenols. Furthermore, there were slightly significant differences between UAE efficiencies at different temperatures as well as amplitudes.

According to the results, the main contributors to the phenolic profiles of the black elderberry press-cake extracts were anthocyanins, cyanidin-3-sambubioside and cyanidin-3-glucoside. These results are similar to those already reported in the literature for elderberry press-cake extracts obtained by ESE using EtOH (90%) + CO_2_ (10%) [39]. Waldbauer et al. (2018) [40] also identified cyanidin-3-sambubioside and cyanidin-3-glucoside as the most abundant phenolic compounds in elderberry press cake, obtained by pressurized liquid extraction (PLE) with 70% MeOH. In our study, the highest concentration for cyanidin-3-sambubioside was 73.24 mg/g DE (678.73 mg/100 g DW), while for cyanidin-3-glucoside, it was 62.22 mg/g DE (571.61 mg/100 g DW), both in MAE-3. Higher recoveries of cyanidin-3-glucoside (0.3–7.5%) and cyanidin-3-sambubioside (0.8–5.5%) were reported in the study conducted by Seabra et al. (2010) [38], where fractionated, high-pressure extractions were used for the treatment of elderberry press cake. This could be explained by the efficient, multistep extraction process for ESEs of elderberry press cake, previously delipidified with supercritical CO_2_, and a prolonged extraction time (100 min in total). However, the differences observed might have also been influenced by the cultivar, location, ripening stage and climatic conditions of the plant material [17].

Rutin is reported to be the major flavanol present in elderberry press cake. The presence of rutin in elderberry press cake was also confirmed by Waldbauer et al. (2018) [40] and Radványi et al. (2013) [37] in PLE extract (70% MeOH, 40 °C) and macerate (50% EtOH, 1 h). In the study by Domínguez et al. (2020) [41], the concentration of rutin (813.08 µg/100 g DW) in UAE elderberry fruit extracts was well below the values obtained in our study. The data reported by Radojković et al. (2021) [42] presented the same trend for elderberry fruit extracts obtained by SLE, MAE and UAE, with a range of concentrations between 5.09 and 8.81 µg/mg DE for rutin and between 0.64 and 0.94 µg/mg DE for chlorogenic acid. Therefore, it seems that MAE is an advantageous alternative to other techniques if the objective is to obtain anthocyanin- and rutin-rich fractions.

### 3.3. Antioxidant and Enzyme Inhibitory Activities

Oxidative stress has been shown to be one of the main causes of the development of diabetes [43,44]. An increase in overcrowding with oxidants promotes the development of diabetes, which is the cause of a greater number of oxidative cells in diabetics than in healthy ones, i.e., higher levels of free radicals [45]. In patients with diabetes, oxidative stress causes changes to two main mechanisms: insulin resistance and secretion [46]. Therefore, our goal was to examine two bioactivities of the extracts, antioxidant and antidiabetic activities, which are closely related. 

To determine the antioxidant activities of black elderberry press-cake extracts, the DPPH assay was applied (Table 4). The UAE extract that proved to be the agent with the best antioxidant activity was UAE-3 (IC_50_ 12.82 μg/mL). A two-fold higher DPPH value was recorded for MAE-3 (IC_50_ 6.89 μg/mL). Overall, MAE extracts proved to exhibit significantly higher antioxidant activity in comparison to others. Their having the highest contents of phenolics identified in MAE extracts was probably what contributed the most to their having the highest antioxidant activities against radical reactive species (a high positive correlation is shown to have been confirmed in Table S1). The antioxidant properties of MAE extracts essentially depended on temperature, these being highest at moderate temperatures (60 and 80 °C). In UAE extracts, higher amplitudes had a negative influence on the observed DPPH values. According to Saebra et al. (2010) [38], the high antioxidant activities of elderberry press-cake extracts may be an indication of the important role of rutin as an antioxidant promoter. Ho et al. (2017) [43] performed a conventional extraction of lyophilized elderberry fruits using EtOH and MeOH, and the highest antioxidant activity reported for MeOH extract (IC_50_ 95.9 μg/mL) was considerably lower in comparison to the activities of all the extracts obtained in this research. 

The inhibition potentials of elderberry press-cake extracts with respect to *α*-amylase are shown in Table 4. The highest inhibition of *α*-amylase was achieved by UAE-1 (IC_50_ 3.98 mg/mL) and MAE-3 (IC_50_ 2.18 mg/mL) (Table 4). In the case of UAEs, a decrease in the inhibition of *α*-amylase activity was observed as the temperature increased from 40 to 60 °C. At higher temperatures, the inhibition activity increased. In MAEs, the extracts obtained at the highest temperature had the lowest *α*-amylase inhibition activities. The ability to inhibit *α*-amylase has been investigated in elderberry fruits [47,48]. According to Ho et al. (2017) [47], the acidic MeOH extract of elderberry fruit was a strong α-amylase inhibitor (IC50 3.9 μg/mL), which inhibitory activity may be ascribed to its high content of polyphenols. The authors also tested cold-pressed elderberry juice for the inhibition of *α*-amylase activity and found that the juice had a higher activity than most of the obtained elderberry extracts. Consequently, a higher concentration of compounds that exert higher activity are to be found in cold-pressed juice, which explains the higher concentrations of elderberry press cake required for the inhibition of *α*-amylase activity.

### 3.4. Principal Component Analysis

**Figure 1:**
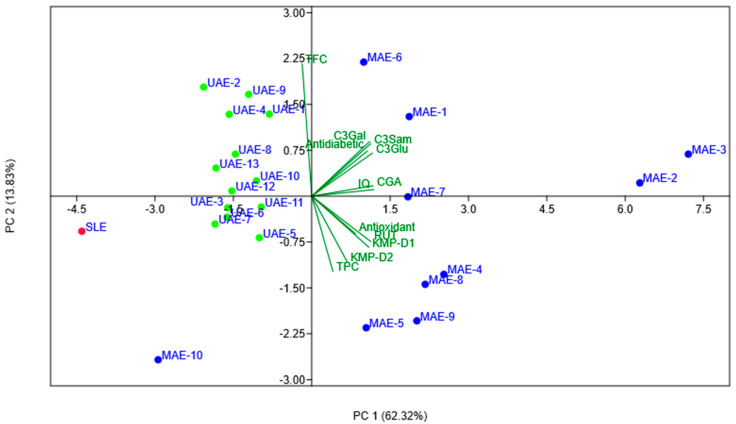
PC1 vs. PC2 biplot of obtained black elderberry press-cake extracts represented as scores and the investigated characteristics as loadings. SLE—red, MAE—blue, UAE—green.

The biplot obtained after performing the PCA, representing both the obtained extracts as scores and the analyzed characteristics as loadings, is shown in Figure 1. The sample obtained using a common SLE is shown in red, the samples obtained under MAE in blue and the samples obtained under UAE in green. 

As indicated by the scree plot shown in Figure S2 (Supplementary Materials), the first two principal components explain 62.32% and 13.83% of the overall data variance, respectively, thus indicating the trustworthiness of the presented PC1 vs. PC2 biplot. The PC1 vs. PC3 and PC2 vs. PC3 biplots, explaining 72.38% and 23.89% of the total variance, respectively, are included in the Supplementary Materials.

The dispersive positions of the obtained sample extracts shown in the PCA biplot (Figure 1) demonstrate the high variability in the compositions of the analyzed polyphenolic compounds and extract properties. The high level of dispersion, indicating high variability in the investigated parameters, was most notable in the samples obtained using MAE. The sample obtained using a common SLE exhibited unique characteristics and is thus isolated in its position in quadrant III of the PCA biplot. All samples obtained using UAE are tightly grouped in quadrants II and III and are thus characterized by similar compositions of polyphenolic compounds. Samples obtained using MAE are the most dispersively distributed, with most of them located in quadrants I and IV. Being positioned in quadrant III, between the SLE and UAE samples, the sample MAE-10 exhibited quite unique properties compared to the other MAE extracts. Samples MAE-1, MAE-2, MAE-3, MAE-6 and MAE-7, obtained under milder temperatures (40, 60 and 80 °C), exhibited positive correlations with both PC1 and PC2, together with the identified polyphenols: isoquercitrin, chlorogenic acid, cyanidin-3-glucoside, cyanidin-3-galactoside and cyanidin-3-sambubioside, which indicates that these extraction conditions are the most effective for obtaining these compounds. On the other hand, explaining less total variability, a PC1 vs. PC3 biplot is less effective in separating obtained samples according to the applied extraction protocols (Figure S3, Supplementary Materials). Explaining only 23.89% of total variability, the PC2 vs. PC3 score plot could not be considered trustworthy enough (Figure S4, Supplementary Materials). As can be seen in the Pearson’s correlation table (Table S1, Supplementary Materials), anthocyanins, cyanidin-3-glucoside, cyanidin-3-galactoside and cyanidin-3-sambubioside had the highest positive correlations with antidiabetic loading (*r* = 0.851, 0.808 and 0.800, respectively; *p* ≤ 0.05), thus indicating the highest antidiabetic properties of the extracts obtained by MAE using lower temperatures. Table S1, therefore, confirms that the antidiabetic feature is to be attributed to the rich content of cyanidin-3-glucoside, cyanidin-3-galactoside and cyanidin-3-sambubioside. The same could be observed in the PCA plot shown in Figure 1. Samples MAE-4, MAE-5, MAE-8 and MAE-9, obtained under higher temperatures of 100 and 120 °C, exhibited positive correlations with PC1 and negative correlations with PC2, together with the polyphenolic compounds rutin and kaempferol derivative 1 and 2, thus indicating their highest abundances in these samples. Furthermore, Figure 1 points to the antioxidant potential of these samples being the highest. However, Table S1 indicates that the compounds isoquercitrin, chlorogenic acid, rutin and kaempferol derivative 1 had the highest positive correlations with the TPC (*r* = 0.364, 0.361, 0.297 and 0.255; *p* ≤ 0.05) and antioxidant properties (*r* = 0.667, 0.661, 0.553, 0.542; *p* ≤ 0.05) of the samples obtained under MAE using higher temperatures. The distribution of the TFC does not follow-up to the distribution of the TPC. The loading TFC exhibited a negative correlation with PC1 and a positive correlation with PC2, similar to the majority of the extracts obtained using UAE, but most specifically UAE-1, UAE-2, UAE-4, and UAE-9, which were obtained under milder temperatures of 40 and 50 °C.

## 4. Conclusions

In this work, the application of two green-based extraction techniques for economically profitable waste management of black elderberry press cake has been reported. The possibility of obtaining high-quality anthocyanin-rich extracts was monitored. The achievement of extracts of the desired quality was determined by the identification and quantification of bioactive compounds, which, owing to their nature, exhibit biological activities, primarily antioxidant and antidiabetic activities. For UAE and MAE assays, the moderate extraction temperature favored EY, TPC and TFC. The highest contents for most of the identified compounds were obtained in MAEs extracted at 80 °C for 5 min (MAE-3), making this technique, under the mentioned conditions, the best for obtaining polyphenolic extracts from black elderberry press cake. This study also enabled the development of an efficient extraction method using mild processing conditions to prevent the degradation of biologically active compounds. According to the PCA, anthocyanins have the highest positive correlations with antidiabetic loading, indicating that the extracts with the highest antidiabetic properties were obtained by MAE using lower temperatures, while isoquercitrin, chlorogenic acid, rutin and kaempferol derivative 1 were found to have the highest positive correlations with antioxidant properties. Despite the fact that studies of *Sambucus* by-products already exist, this is the first report to give an overview of the polyphenolic profile of black elderberry press-cake extracts with antioxidant and antidiabetic properties. This analysis was carried out using solvents and techniques considered “green” in the food and pharmaceutical industries and which are efficient in terms of economic viability and feasibility due to significantly shorter extraction times, which represents the most important contribution of this work.

## Figures and Tables

**Table 1:** EY, TPC and TFC values obtained for black elderberry press cake using different extraction techniques under different extraction conditions.

Extraction	Samples	Extraction Conditions	EY * (%)	TPC * (mg GAE/g DE)	TFC * (mg CE/g DE)
SLE	SLE-1	30% EtOH, room temperature, 24 h	26.58 ± 0.24 ^d^^–g^	150.50 ± 5.96 ^cde^	48.52 ± 1.75 ^hij^
	SLE-2	50% EtOH, room temperature, 24 h	15.48 ± 0.71 ^kl^	146.02 ± 8.27 ^c^^–f^	49.04 ± 3.23 ^g^^–j^
	SLE-3	70% EtOH, room temperature, 24 h	12.84 ± 0.14 ^m^	141.77 ± 8.00 ^d^^–h^	49.51 ± 3.67 ^f^^–j^
UAE	UAE-1	30% EtOH, 40 °C, A 20%, 11 min	19.68 ± 0.90 ^i^	134.41 ± 0.16 ^f^^–j^	55.84 ± 0.37 ^a^^–d^
	UAE-2	30% EtOH, 50 °C, A 20%, 19 min	28.48 ± 0.89 ^bcd^	122.38 ± 3.08 ^j^	57.98 ± 0.76 ^ab^
	UAE-3	30% EtOH, 60 °C, A 20%, 40 min	28.02 ± 0.50 ^cde^	128.07 ± 3.04 ^ij^	51.46 ± 0.52 ^c-i^
	UAE-4	30% EtOH, 40 °C, A 60%, 2 min	26.06 ± 0.79 ^fgh^	129.30± 1.08 ^hij^	56.39 ± 1.45 ^abc^
	UAE-5	30% EtOH, 50 °C, A 60%, 4 min	26.40 ± 0.42 ^e^^–h^	132.95 ± 0.86 ^g^^–j^	48.65 ± 0.49 ^g^^–j^
	UAE-6	30% EtOH, 60 °C, A 60%, 7 min	29.04 ± 1.24 ^bc^	143.23 ± 2.57 ^c^^–h^	53.11 ± 1.44 ^b^^–g^
	UAE-7	30% EtOH, 70 °C, A 60%, 13 min	30.16 ± 0.60 ^ab^	136.48 ± 1.84 ^e^^–j^	50.12 ± 0.52 ^e^^–j^
	UAE-8	30% EtOH, 80 °C, A 60%, 23 min	28.18 ± 0.51 ^cde^	132.85 ± 1.40 ^g^^–j^	56.56 ± 0.66 ^abc^
	UAE-9	30% EtOH, 40 °C, A 100%, 1 min	24.50 ± 0.93 ^h^	134.55 ± 0.66 ^f^^–j^	58.39 ± 0.44 ^a^
	UAE-10	30% EtOH, 50 °C, A 100%, 2 min	25.56 ± 0.63 ^gh^	130.51 ± 3.17 ^g^^–j^	51.44 ± 0.39 ^c^^–i^
	UAE-11	30% EtOH, 60 °C, A 100%, 4 min	27.90 ± 0.18 ^c^^–f^	138.75 ± 4.16 ^e^^–i^	51.72 ± 0.57 ^c^^–i^
	UAE-12	30% EtOH, 70 °C, A 100%, 6 min	29.44 ± 0.91 ^abc^	125.58 ± 2.12 ^ij^	50.99 ± 0.89 ^d^^–i^
	UAE-13	30% EtOH, 80 °C, A 100%, 10 min	31.28 ± 1.09 ^a^	130.64 ± 2.42 ^g^^–j^	53.98 ± 0.61 ^a^^–f^
MAE	MAE-1	30% EtOH, 40 °C, 5 min	13.33 ± 0.01 ^m^	156.08 ± 2.50 ^bc^	52.26 ± 0.90 ^c^^–h^
	MAE-2	30% EtOH, 60 °C, 5 min	8.50 ± 0.30 ^n^	166.02 ± 3.31 ^ab^	54.30 ± 1.40 ^a^^–e^
	MAE-3	30% EtOH, 80 °C, 5 min	9.27 ± 0.01 ^n^	122.00 ± 4.80 ^j^	40.29 ± 0.59 ^m^
	MAE-4	30% EtOH, 100 °C, 5 min	17.27 ± 0.50 ^jk^	154.07 ± 5.24 ^bcd^	45.33 ± 0.93 ^j^^–m^
	MAE-5	30% EtOH, 120 °C, 5 min	18.80 ± 0.31 ^ij^	150.90 ± 5.16 ^cde^	48.06 ± 1.19 ^h^^–k^
	MAE-6	30% EtOH, 40 °C, 10 min	14.41 ± 0.24 ^lm^	123.77 ± 6.29 ^ij^	42.32 ± 0.92 ^lm^
	MAE-7	30% EtOH, 60 °C, 10 min	16.53 ± 0.34 ^k^	157.92 ± 2.44 ^abc^	47.75 ± 0.51 ^h^^–k^
	MAE-8	30% EtOH, 80 °C, 10 min	16.57 ± 0.17 ^k^	171.86 ± 4.51 ^a^	50.92 ± 0.73 ^d^^–i^
	MAE-9	30% EtOH, 100 °C, 10 min	20.63 ± 0.20 ^i^	137.90 ± 2.70 ^e^^–i^	43.15 ± 1.01 ^klm^
	MAE-10	30% EtOH, 120 °C, 10 min	25.77 ± 0.28 ^gh^	145.17 ± 6.53 ^c^^–g^	46.50 ± 0.65 ^i^^–j^

* Different letters within a column indicate a significant difference between samples at *p* < 0.05. MAE—microwave-assisted extraction, UAE—ultrasound-assisted extraction, EY—extraction yield, TPC—total phenolic content, TFC—total flavonoid content, EtOH—ethanol, A—amplitude.

**Table 2:** Characterized compounds in black elderberry press-cake extracts obtained by different extraction techniques using HPLC-MS/MS.

Peak	Rt (min)	[M − H]^−^ (*m*/*z*)	[M + H]^+^ (*m*/*z*)	MS^2^ (*m*/*z*)	MS^3^ (*m*/*z*)	Compounds **
1	6.55	373		355(100), 343(80), 193(33)	193(100)	Ferulic acid hexoside derivative 1 *
2	9.24	153		123(100), 109(27)		Protocatechuic acid
3	9.46		449	287(100)		Cyanidin-3-galactoside
4	11.03		581	287(100)		Cyanidin-3-sambubioside
5	11.14		449	287(100)		Cyanidin-3-glucoside
6	13.14	371		325(100), 163(51)		*p*-coumaric acid hexoside derivative *
7	14.86	353		191(100), 179(5)		Chlorogenic acid (3-caffeoylquinic acid)
8	15.40	597		285(100), 241(9)		Kaempferol derivative 1 *
9	15.42		599	581(100)	287(100)	Cyanidin-3-sambubioside derivative
10	16.07	465		285(100), 241(11)		Kaempferol derivative 2 *
12	17.12	571		487(100)	337(100), 355(48), 193(10)	Ferulic acid hexoside derivative 2 *
12	17.73	755		300(100), 591(83), 301(44), 271(19)		Quercetin trisaccharide *
13	19.07	609		301(100), 300(23), 179(3)		Rutin
14	19.63	463		301(100), 300(16), 179(1)		Isoquercitrin
15	19.77	593		285(100)	257(100), 267(44), 241(38), 229(37)	Kaempferol-3-rutinoside
16	20.65	549		531(100), 353(94)	353(100)	Dicaffeoylquinic acid derivative *

* Compound identified in black elderberry press cake for the first time. ** All compounds were identified in all of the extracts obtained.

**Table 3:** Contents of phenolic compounds in black elderberry press-cake extracts determined by HPLC-DAD.

Samples	C3Gal */** (mg/g DE)	C3Glu */** (mg/g DE)	C3Sam */**/*** (mg/g DE)	RUT */** (mg/g DE)	IQ */** (mg/g DE)	KMP-D1 */**/**** (mg/g DE)	KMP-D2 */**/**** (mg/g DE)	CGA */** (mg/g DE)
SLE-1	1.47 ± 0.04 ^n^	12.41 ^n^ ± 0.19 ^n^	17.64 ± 0.26 ^l^	4.17 ± 0.12 ^n^	2.72 ± 0.08 ^l^	1.34 ± 0.01 ^m^	0.25 ± 0.01 ^n^	1.99 ± 0.04 ^m^
UAE-1	2.54 ± 0.05 ^g^	25.18 ± 0.38 ^hi^	33.67 ± 0.51 ^ef^	7.79 ± 0.23 ^jk^	3.77 ± 0.08 ^f^	3.40 ± 0.08 ^k^	0.67 ± 0.01 ^klm^	2.85 ± 0.04 ^h^
UAE-2	2.28 ± 0.05 ^ij^	22.59 ± 0.34 ^k^	30.22 ± 0.45 ^gh^	7.19 ± 0.22 ^kl^	2.92 ± 0.06 ^jkl^	3.30 ± 0.05 ^kl^	0.69 ± 0.02 ^jkl^	2.10 ± 0.01 ^j^^–m^
UAE-3	1.96 ± 0.06 ^m^	21.68 ± 0.33 ^kl^	26.19 ± 0.39 ^jk^	8.04 ± 0.24 ^ij^	3.01 ± 0.09 ^ijk^	3.75 ± 0.09 ^j^	0.69 ± 0.01 ^jkl^	2.11 ± 0.02 ^j^^–m^
UAE-4	2.37 ± 0.07 ^hi^	22.62 ± 0.34 ^k^	31.75 ± 0.48 ^fg^	6.73 ± 0.20 ^l^	3.13 ± 0.09 ^hij^	3.03 ± 0.05 ^l^	0.70 ± 0.01 ^jk^	2.20 ± 0.04 ^jkl^
UAE-5	2.41 ± 0.07 ^ghi^	26.59 ± 0.40 ^gh^	35.04 ±0.53 ^de^	8.47 ± 0.25 ^hi^	3.23 ± 0.06 ^ghi^	3.93 ± 0.08 ^ij^	0.77 ± 0.02 ^ghi^	2.21 ± 0.00 ^jk^
UAE-6	2.19 ± 0.04 ^jkl^	22.77 ± 0.34 ^jk^	28.34 ± 0.43 ^hi^	8.86 ± 0.27 ^fgh^	2.89 ± 0.06 ^kl^	4.17 ± 0.06 ^f^^–i^	0.66 ± 0.02 ^klm^	2.10 ± 0.01 ^j^^–m^
UAE-7	2.20 ± 0.07 ^jk^	19.63 ± 0.29 ^m^	24.99 ± 0.37 ^jk^	9.03 ± 0.27 ^fgh^	2.81 ± 0.08 ^kl^	4.28 ± 0.05 ^fg^	0.65 ± 0.01 ^lm^	2.07 ± 0.02 ^lm^
UAE-8	2.08 ± 0.06 ^klm^	21.59 ± 0.32 ^kl^	25.98 ± 0.39 ^jk^	9.09 ± 0.27 ^fg^	3.04 ± 0.09 ^ijk^	4.28 ± 0.07 ^fg^	0.73 ± 0.01 ^ij^	2.16 ± 0.03 ^jkl^
UAE-9	2.47 ± 0.07 ^gh^	24.49 ± 0.37 ^ij^	33.82 ± 0.51 ^ef^	4.82 ± 0.14 ^m^	3.42 ± 0.10 ^g^	3.27 ± 0.01 ^kl^	0.80 ± 0.01 ^fg^	2.40 ± 0.05 ^i^
UAE-10	2.44 ± 0.05 ^gh^	27.27 ± 0.41 ^g^	34.93 ± 0.52 ^de^	8.70 ± 0.26 ^gh^	3.35 ± 0.09 ^gh^	4.04 ± 0.06 ^ghi^	0.81 ± 0.00 ^fg^	2.38 ± 0.03 ^i^
UAE-11	2.36 ± 0.07 ^hi^	26.75 ± 0.40 ^gh^	32.65 ± 0.49 ^f^	9.78 ± 0.29 ^e^	3.18 ± 0.06 ^hi^	4.65 ± 0.11 ^e^	0.80 ± 0.01 ^fg^	2.23 ± 0.00 ^j^
UAE-12	2.20 ± 0.07 ^jk^	21.67 ± 0.33 ^kl^	26.66 ± 0.40 ^ij^	8.99 ± 0.27 ^fgh^	3.02 ± 0.03 ^ijk^	4.27 ± 0.06 ^fgh^	0.69 ± 0.02 ^jkl^	2.08 ± 0.06 ^klm^
UAE-13	2.09 ± 0.06 ^klm^	19.86 ± 0.30 ^lm^	24.55 ± 0.37 ^jk^	9.07 ± 0.27 ^fgh^	2.88 ± 0.06 ^kl^	4.33 ± 0.09 ^f^	0.75 ± 0.01 ^hi^	2.00 ± 0.07 ^m^
MAE-1	2.92 ± 0.06 ^de^	42.38 ± 0.64 ^bc^	54.15 ± 0.81 ^b^	9.47 ± 0.28 ^ef^	5.12 ± 0.08 ^c^	3.99 ± 0.01 ^hij^	0.79 ± 0.01 ^fgh^	4.03 ± 0.07 ^c^
MAE-2	3.98 ± 0.08 ^b^	60.99 ± 0.92 ^a^	73.22 ± 1.10 ^a^	16.60 ± 0.50 ^b^	8.04 ± 0.16 ^a^	7.11 ± 0.16 ^b^	0.70 ± 0.01 ^jk^	6.17 ± 0.12 ^a^
MAE-3	4.15 ± 0.12 ^a^	62.22 ± 0.93 ^a^	73.24 ± 1.10 ^a^	18.16 ± 0.54 ^a^	7.41 ± 0.22 ^b^	8.06 ± 0.23 ^a^	1.02 ± 0.02 ^c^	5.91 ± 0.01 ^b^
MAE-4	3.10 ± 0.09 ^c^	39.92 ± 0.60 ^de^	47.84 ± 0.72 ^c^	12.96 ± 0.39 ^c^	4.58 ± 0.14 ^de^	6.02 ± 0.11 ^c^	0.96 ± 0.01 ^d^	3.47 ± 0.01 ^e^
MAE-5	2.04 ± 0.04 ^lm^	19.10 ± 0.29 ^m^	24.47 ± 0.37 ^k^	12.88 ± 0.39 ^c^	4.56 ± 0.14 ^de^	6.06 ± 0.03 ^c^	1.10 ± 0.01 ^b^	3.32 ± 0.01 ^f^
MAE-6	2.83 ± 0.06 ^ef^	41.25 ± 0.62 ^cd^	52.79 ± 0.79 ^b^	9.47 ± 0.28 ^ef^	4.72 ± 0.12 ^d^	4.04 ± 0.04 ^ghi^	0.63 ± 0.00 ^m^	3.67 ± 0.01 ^d^
MAE-7	3.05 ± 0.09 ^cd^	43.80 ± 0.66 ^b^	54.73 ± 0.82 ^b^	10.44 ± 0.31 ^d^	4.43 ± 0.13 ^e^	4.66 ± 0.06 ^e^	0.75 ± 0.02 ^hi^	3.35 ± 0.01 ^ef^
MAE-8	2.78 ± 0.08 ^ef^	38.81 ± 0.58 ^e^	46.12 ± 0.69 ^c^	12.36 ± 0.37 ^c^	4.59 ± 0.14 ^de^	5.68 ± 0.06 ^d^	0.88 ± 0.01 ^e^	3.45 ± 0.00 ^ef^
MAE-9	2.70 ± 0.08 ^f^	30.46 ± 0.46 ^f^	36.75 ± 0.55 ^d^	12.81 ± 0.38 ^c^	4.36 ± 0.13 ^e^	6.10 ± 0.18 ^c^	1.21 ± 0.04 ^a^	3.02 ± 0.07 ^g^
MAE-10	0.58 ± 0.02 ^o^	3.25 ± 0.05 ^o^	4.51 ± 0.07 ^m^	8.56 ± 0.26 ^ghi^	3.17 ± 0.11 ^hi^	4.10 ± 0.00 ^f^^–i^	0.82 ± 0.02 ^f^	2.38 ± 0.02 ^i^

* C3Gal—cyanidin-3-O-galactoside, C3Glu—cyanidin-3-O-glucoside, C3Sam—cyanidin-3-O-sambubioside; RUT—rutin; IQ—isoquercitrin, KMP-D1—kaempferol derivate 1, KMP-D2—kaempferol derivate 2, CGA—chlorogenic acid. ** Different letters within a column indicate a significant difference between samples at *p* < 0.05. *** Expressed as C3Glu. **** Expressed as kaempferol.

**Table 4:** Antioxidant activities and *α*-amylase inhibitory activities of black elderberry press-cake extracts.

Samples	DPPH * IC_50_ (μg/mL)	*A*-amylase * IC_50_ (mg/mL)
SLE-1	14.96 ± 0.77 ^b^^–^	6.88 ± 0.68 ^a^
UAE-1	14.67 ± 1.39 ^b^^–e^	3.98 ± 0.21 ^d^^–g^
UAE-2	16.20 ± 1.33 ^a^^–d^	4.64 ± 0.25 ^b^^–e^
UAE-3	12.82 ± 0.72 ^d^^–g^	4.63 ± 0.45 ^b^^–e^
UAE-4	14.06 ± 0.95 ^b^^–e^	4.45 ± 0.31 ^b^^–f^
UAE-5	14.63 ± 1.31 ^b^^–e^	4.91 ± 0.29 ^bcd^
UAE-6	14.46 ± 0.94 ^b^^–e^	5.08 ± 0.45 ^bc^
UAE-7	16.65 ± 1.32 ^abc^	4.79 ± 0.25 ^b^^–e^
UAE-8	14.85 ± 1.46 ^b^^–e^	4.52 ± 0.41 ^b^^–f^
UAE-9	13.51 ± 0.88 ^c^^–f^	4.18 ± 0.22 ^b^^–f^
UAE-10	18.62 ± 1.59 ^a^	4.47 ± 0.44 ^b^^–f^
UAE-11	17.29 ± 1.49 ^ab^	4.54 ± 0.25 ^b^^–f^
UAE-12	16.37 ± 1.55 ^a^^–d^	4.31 ± 0.37 ^b^^–f^
UAE-13	18.74 ± 1.69 ^a^	4.32 ± 0.41 ^b^^–f^
MAE-1	12.09 ± 1.01 ^e^^–h^	3.00 ± 0.20 ^gh^
MAE-2	7.88 ± 0.69 ^i^	2.47 ± 0.13 ^h^
MAE-3	6.89 ± 0.64 ^i^	2.18 ± 0.17 ^h^
MAE-4	11.97 ± 1.09 ^e^^–h^	3.57 ± 0.24 ^fg^
MAE-5	13.33 ± 1.28 ^c^^–f^	3.75 ± 0.28 ^efg^
MAE-6	10.45 ± 0.92 ^f^^–i^	3.81 ± 0.21 ^efg^
MAE-7	8.60 ± 0.63 ^hi^	4.04 ± 0.27 ^c^^–g^
MAE-8	9.45 ± 0.86 ^ghi^	4.08 ± 0.38 ^c^^–f^
MAE-9	7.80 ± 0.76 ^i^	3.92 ± 0.23 ^d^^–g^
MAE-10	10.43 ± 1.02 ^f^^–i^	5.14 ± 0.40 ^b^

* Different letters within a column indicate a significant difference between samples at *p* < 0.05.

## Data Availability

Not applicable.

## Supplementary Materials

The following supporting information can be downloaded at: https://www.mdpi.com/article/10.3390/biology11101465/s1, Figure S1: HPLC profile of black elderberry press-cake SLE extract recorded at (a) 290 nm, (b) 350 nm and (c) 520 nm; Figure S2: PCA scree plot; Figure S3: PC1 vs. PC3 biplot of obtained black elderberry press-cake extracts represented as scores with investigated characteristics as loadings (SLE—red, MAE—blue, UAE—green); Figure S4: PC2 vs. PC3 biplot of obtained black elderberry press-cake extracts represented as scores with investigated characteristics as loadings (SLE—red, MAE—blue, UAE—green); Table S1: Pearson’s correlation coefficients.
