# Supplementary material for: Black Elderberry Press Cake as a Source of Bioactive Ingredients Using Green-Based Extraction Approaches
Source: Biology (Basel). 2022 Oct 6;11(10):1465. doi: 10.3390/biology11101465 (PMC9598939; doi:10.3390/biology11101465)
Supplement: Supplementary file 1 [file biology-11-01465-s001.zip › biology-1911920-supplementary.pdf]

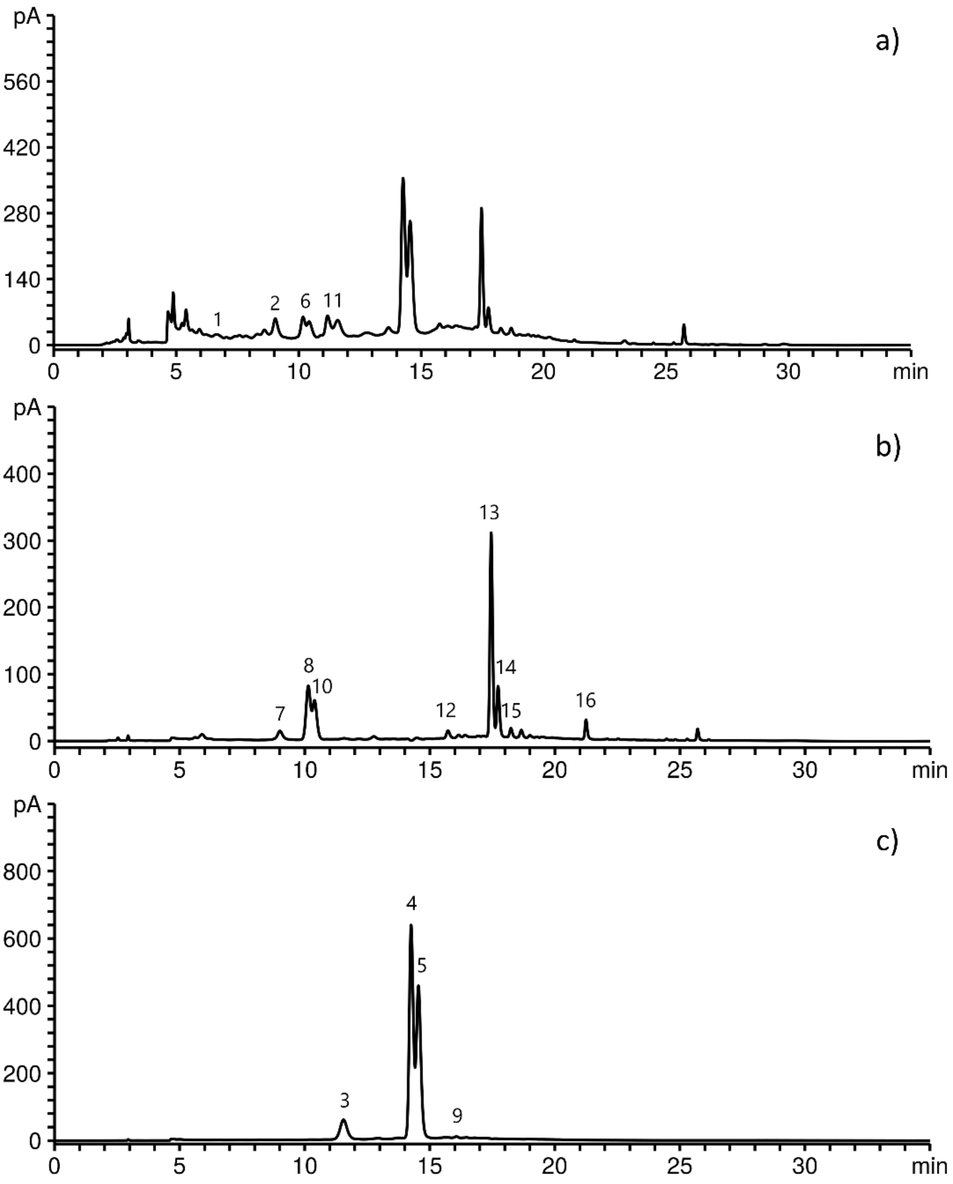

Figure S1. HPLC profile of black elderberry press cake SLE extract recorded at a) 290 nm, b) 350 nm and c) 520 nm.

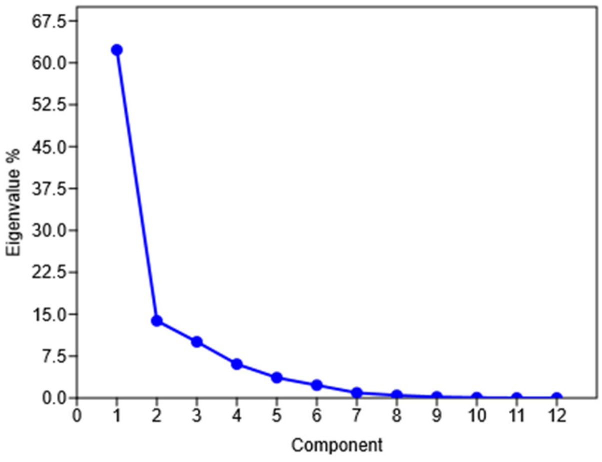

Figure S2. PCA scree plot.

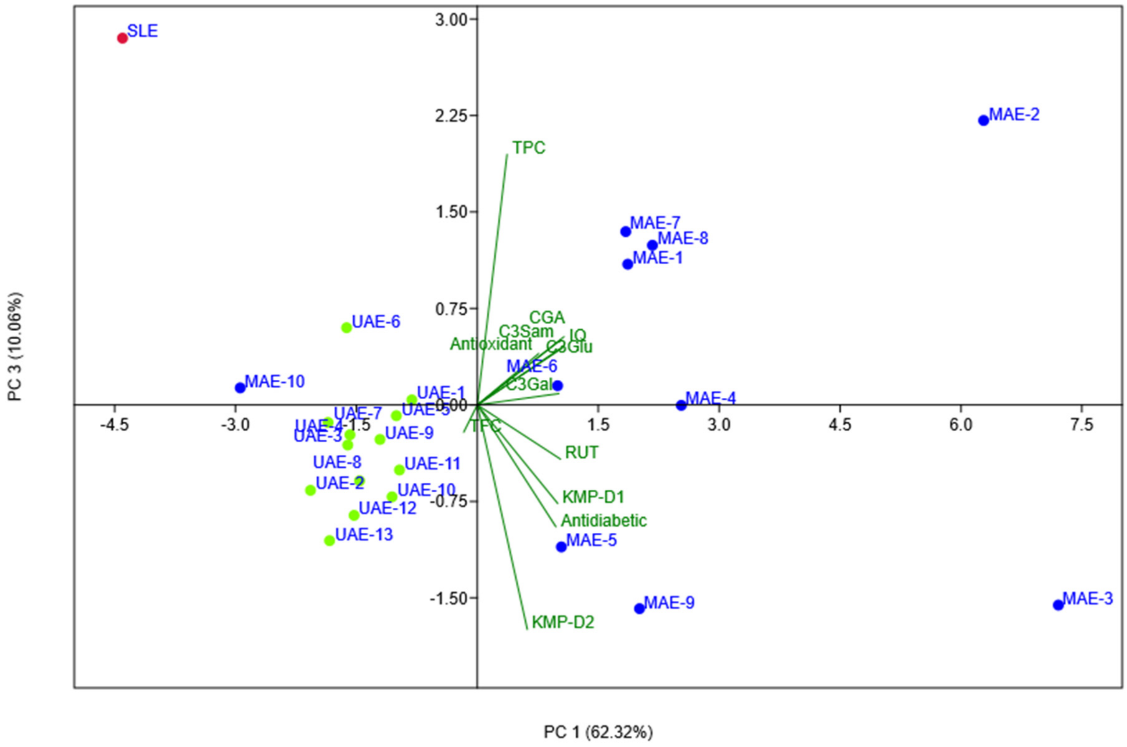

Figure S3. PC1 *vs* PC3 bi-plot of obtained black elderberry press cake extracts represented as scores and investigated characteristics as loadings (SLE – red; MAE – blue; UAE – green).

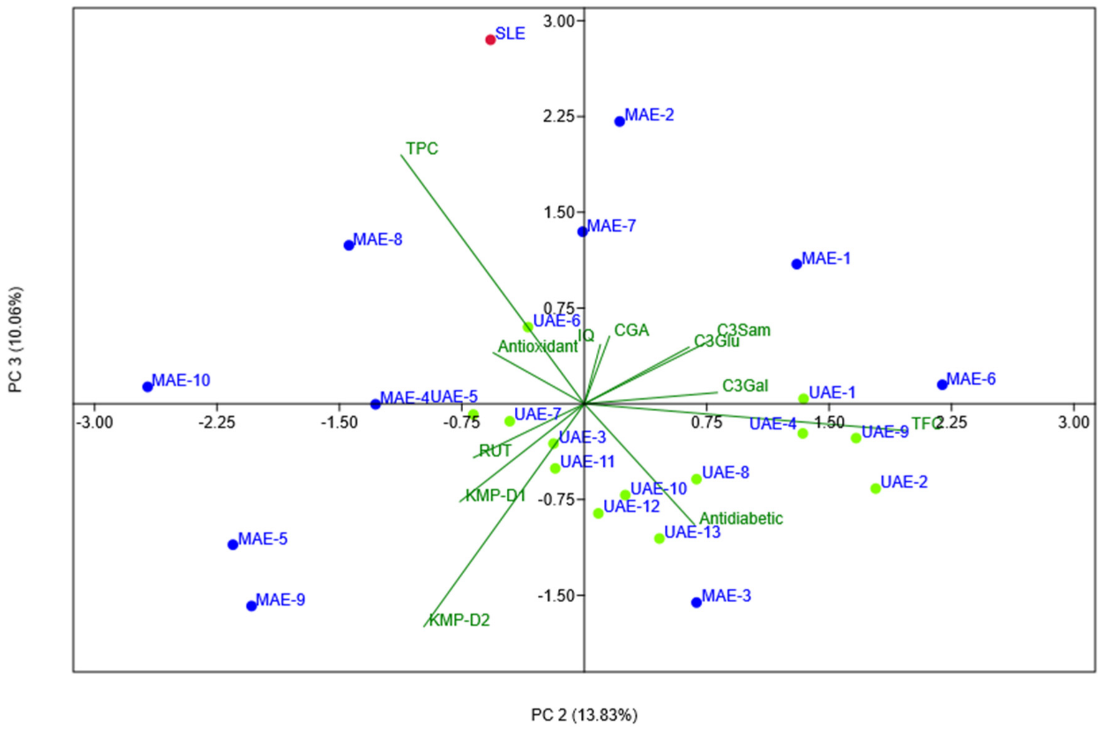

Figure S4. PC2 *vs* PC3 bi-plot of obtained black elderberry press cake extracts represented as scores and investigated characteristics as loadings (SLE – red; MAE – blue; UAE – green).

Table S1. Pearson's correlation coefficients.

	**TPC**	**TFC**	**AO**	**AD**	**C3Gal**	**C3Sam**	**C3Glu**	**RUT**	**IQ**	**KMP-D1**	**KMP-D2**	**CGA**
**TPC**												
**TFC**	-0.319											
**AO**	0.368	-0.131										
**AD**	-0.026	0.122	0.421									
**C3Gal**	0.153	0.043	0.424	0.851								
**C3Sam**	0.249	0.051	0.521	0.800	0.965							
**C3Glu**	0.264	-0.004	0.533	0.808	0.963	0.995						
**RUT**	0.297	-0.375	0.553	0.728	0.701	0.695	0.748					
**IQ**	0.364	-0.104	0.667	0.762	0.817	0.875	0.890	0.843				
**KMP-D1**	0.255	-0.353	0.542	0.736	0.664	0.632	0.688	0.977	0.782			
**KMP-D2**	0.074	-0.219	0.464	0.576	0.315	0.251	0.284	0.631	0.378	0.736		
**CGA**	0.361	-0.085	0.662	0.755	0.808	0.876	0.89	0.829	0.996	0.761	0.343	
